# Analgesic Effect of Acupuncture Is Mediated via Inhibition of JNK Activation in Astrocytes after Spinal Cord Injury

**DOI:** 10.1371/journal.pone.0073948

**Published:** 2013-09-09

**Authors:** Jee Y. Lee, Doo C. Choi, Tae H. Oh, Tae Y. Yune

**Affiliations:** 1 Age-Related and Brain Diseases Research Center, Kyung Hee University, Seoul, Korea; 2 Neurodegeneration Control Research Center, Kyung Hee University, Seoul, Korea; 3 Department of Biochemistry and Molecular Biology, School of Medicine, Kyung Hee University, Seoul, Korea; Boston Children’s Hospital and Harvard Medical School, United States of America

## Abstract

Acupuncture (AP) has been used worldwide to relieve pain. However, the mechanism of action of AP is poorly understood. Here, we found that AP relieved neuropathic pain (NP) by inhibiting Jun-N-terminal kinase (JNK) activation in astrocytes after spinal cord injury (SCI). After contusion injury which induces the below-level (L4-L5) NP, Shuigou (GV26) and Yanglingquan (GB34) acupoints were applied. At 31 d after injury, both mechanical allodynia and thermal hyperalgesia were significantly alleviated by AP applied at GV26 and GB34. Immunocytochemistry revealed that JNK activation was mainly observed in astrocytes after injury. AP inhibited JNK activation in astrocytes at L4-L5 level of spinal cord. The level of p-c-Jun known, a downstream molecule of JNK, was also decreased by AP. In addition, SCI-induced GFAP expression, a marker for astrocytes, was decreased by AP as compared to control groups. Especially, the number of hypertrophic, activated astrocytes in laminae I–II of dorsal horn at L4-5 was markedly decreased by AP treatment when compared with vehicle and simulated AP-treated groups. When animals treated with SP600125, a specific JNK inhibitor, after SCI, both mechanical allodynia and thermal hyperalgesia were significantly attenuated by the inhibitor, suggesting that JNK activation is likely involved in SCI-induced NP. Also, the expression of chemokines which is known to be mediated through JNK pathway was significantly decreased by AP and SP600125 treatment. Therefore, our results indicate that analgesic effect of AP is mediated in part by inhibiting JNK activation in astrocytes after SCI.

## Introduction

Neuropathic pain (NP) is one of the pathological pains which are caused primarily by damage of the peripheral or central nervous system (CNS) [1]. NP includes spontaneous burning pain or stimulus-evoked pain which is represented by hyperalgesia evoked by noxious stimuli and allodynia evoked by a non-noxious stimuli [2]. A majority of spinal cord injury (SCI) patients are known to experience central NP. SCI-induced NP can be localized above-, at-, and below-levels as rostral, same and caudal position from the injury site [3–5]. However, currently available treatments for the SCI-induced NP are only partially effective, and additional therapeutic development for this NP is hindered by our incomplete understanding of how neuropathic pain is induced and maintained.

Increasing evidences show that after SCI, mitogen activated protein kinase (MAPK) including p38MAPK, extracellular signal-regulated kinase (ERK) and c-Jun N-terminal kinase (JNK) are activated in glial cells and play a pivotal role in the induction and maintenance of central and peripheral NP [6–11]. For example, both peripheral nerve injury and SCI induce p38MAPK and ERK activation in microglia in the spinal cord [6–8,12,13]. Our recent report also shows that an intrathecal injection of p38MAPK inhibitor (SB203580) or ERK inhibitor (PD98059) after SCI attenuates mechanical allodynia and hyperalgesia [14]. Furthermore, PGE2 produced via ERK-dependent signaling in activated microglia mediates SCI-induced NP through EP2, PGE2 receptor, expressed in spinal cord neurons [8].

It has been shown that JNK is persistently activated in astrocytes in the spinal cord after pheripheral nerve injury [9,15–17]. Administration of JNK inhibitors such as SP600125 and D-JNKI-1 also alleviates sciatic nerve ligation (SNL)-induced NP [9,18]. Recent evidence also shows that JNK induces expression of CCL2/MCP-1 (monocyte chemoattractant protein-1) chemokine in spinal cord astrocytes, which contributes to central sensitization and NP facilitation by enhancing excitatory synaptic transmission [16]. Although JNK activation after SCI has been known to be involved in apoptotic neuronal cell death and axonal degeneration, leading to limiting motor recovery after SCI [19–22], the role of JNK activation in the development or maintenance of chronic NP after injury has not been examined yet.

Acupuncture (AP) is known to relieve peripheral NP as well as acute or chronic inflammatory pain via inhibition of microglial activation and production of inflammatory mediators in animal models [23–25]. In clinical trials, AP is also shown to relieve chronic lower back, arthritic pain [23,26], and NP following the CNS injuries including SCI [27,28]. However, the precise mechanism of action of AP on NP is not fully understood. In this regard, our recent study [14] shows that AP relieves SCI-induced NP at below-level by inhibiting reactive oxygen species (ROS)-induced p38MAPK and ERK activation in microglia. Since JNK activation is known to be involved in pheripheral nerve injury-induced NP [9], we tested a hypothesis that AP would relieve NP by influencing JNK signaling after SCI. We found that AP relieved the below level NP by inhibiting JNK activation in astrocytes after injury.

## Materials and Methods

### Ethics Statement

All surgical interventions and postoperative animal care were approved by the Animal Care Committee of the Kyung Hee University.

### Spinal cord injury

Adult rats [Sprague Dawley, male, 250-300 g; Sam: TacN (SD) BR; Samtako, Osan, Korea] were maintained under a constant temperature (23 ± 1 °C) and humidity (60 ± 10%) under a 12 h light/ dark cycle (light on 07:30–19:30 h) with *ad libitum* access to drinking water and food. Prior to surgery, rats were weighed and anesthetized with chloral hydrate (500 mg/kg intraperitoneal injection). An adequate level of anesthesia was determined by monitoring the corneal and hindlimb withdrawal reflexes. The back and neck regions were then shaved and laminectomy was performed at the T9-T10 level, exposing the cord beneath without disrupting the dura. The spinous processes of T8 and T11 were then clamped to stabilize the spine, and the exposed dorsal surface of the cord was subjected to moderate contusion injury (10 g x 25 mm) using a New York University (NYU) impactor as described previously [29]. For the sham-operated controls, the animals underwent a T9-T10 laminectomy without weight-drop injury. Throughout the surgical procedure, body temperature was maintained at 37 ± 0.5 °C with a heating pad (Biomed S.L., Alicante, Spain). After the injury the muscles and skin were closed in layers, and the rats were placed in a temperature and humidity-controlled chamber overnight. Postoperatively, rats were received subcutaneously supplemental fluids (5 ml, lactated ringer) and antibiotics (gentamicin, 5 mg/kg intramuscular injection) once daily for 5 d after surgery. The rats were housed one per cage after injury with water and food easily accessible. Body weights and the remaining chow and water weight were recorded each morning for all animals. The bladder was emptied manually three times per day until reflexive bladder emptying was established.

### Acupuncture treatment

**Figure 1:**
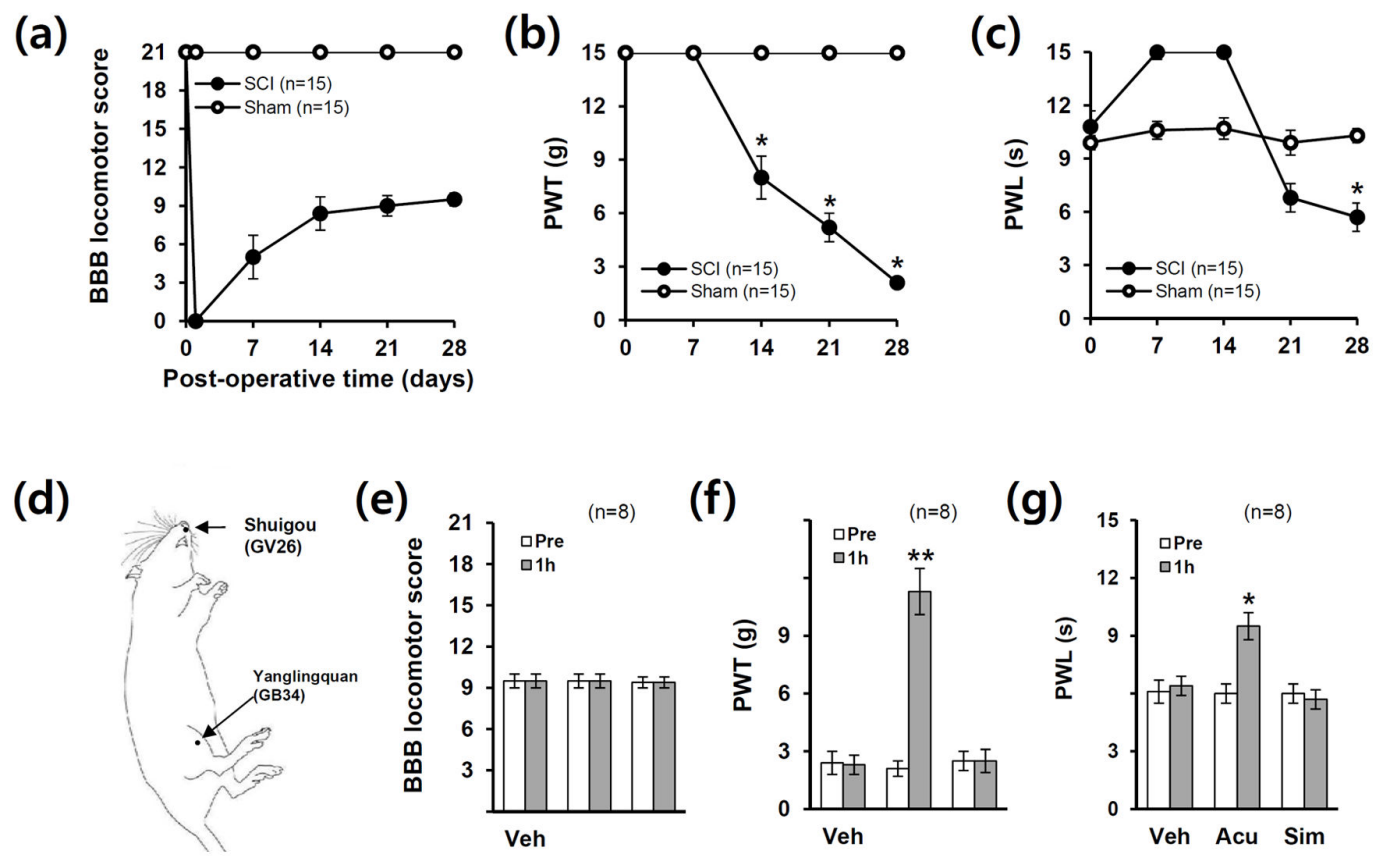
Acupuncture relieves neuropathic pain after SCI. (a) Hind limb locomotor function as assessed by BBB scores after SCI. *p < 0.05. (b–c) Pain responses to mechanical stimuli (PWT) and heat stimuli (PWL) after injury (n = 15). Data (a-c) are presented as mean ± SEM (*p < 0.05 vs sham, df = 1, repeated measures ANOVA) (d) Schematic diagram showing acupoints applied to SCI animals. Acupuncture was applied at two specific acupoints, Shuigou (GV26) and Yanglingquan (GB34), throughout experiments. (e) There was no significant difference in BBB scores in all groups (n = 8). AP treatment significantly reduced mechanical allodynia (f) and heat hyperalgesia (g) when compared with those in the vehicle (Veh) or simulated AP (Sim) control after injury (n = 8). Note that simulated AP had no significant effect on pain relief. Data (e-g) are presented as mean ± SEM (*p < 0.05, **p < 0.01 vs Pre (before treatment); unpaired Student’s t test).

To establish homogenous experimental groups, first, we selected those animals with 9-10 BBB scores at 28 d after SCI (similar motor function improvement). Second, we selected only animals that chronic neuropathic pain was developed (mechanical allodyna, PWT; 1.5-3 g and heat sensitivity, PWL; 5.5-6.5 s), and then selected rats were divided randomly into each experimental group including vehicle, AP and simulated AP (control) treatments. More than 80% of rats were in these criteria and rats not satisfying these criteria were excluded. Since our recent report showed that AP applied at both Shuigou (GV26) and Yanglingquan (GB34) acupoints simultaneously exerts an analgesic effect against SCI-induced NP at below level [14], AP was applied at both GV26 and GB34 without anesthesia (Figure 1d) using an immobilization apparatus designed by our laboratory (Figure S1) [14,30]. GB34 is located at the point of intersection of lines from the anterior border to the head of the fibula, and GV26, located at the mid points between base of the columnar nasi and the upper lip, on the facial midline [31] (Figure 1A). Stainless-steel AP needles of 0.20 mm in diameter were inserted to a depth of 4-6 mm at each acupoint bilaterally, turned at a rate of two spins per second for 30 s, and then the needles were retained for 30 min. AP treatment was applied at POD 31 and pain behavioral tests were performed at 1 h to 4 h after AP treatment. We used rats received injury without any AP treatment as a vehicle control. For another control experiment, a simulated AP treatment with a toothpick at each acupoint was also performed as described [14,30,32]. In brief, the skin of each specific acupoint was tapped with the tip of a toothpick to imitate an AP needle insertion. The acupiont was then gently touched with the tip of a toothpick, and the toothpick was turned at a rate of two spins per second for 30 s. After 30 minutes, to simulate withdrawal of the needle, a toothpick momentarily touched the skin of the acupoint and was then quickly pulled away [14,30,32].

### Pain behavioral tests

All pain behavioral testing was performed by trained investigators who were blind as to the experimental conditions and began at postoperative days (POD) 28 to confirm behavioral signs of SCI-induced chronic NP before AP or drug treatment as our previous report [14]. For all experiments, we used only animals that chronic NP following SCI was developed. At POD 28, hindlimb locomotion of injured animals were recovered well enough to yield reliable withdrawal reflex measures as described previously [14,33].

Mechanical allodynia was assessed by the paw withdrawal threshold (PWT) in response to probing with a series of calibrated von Frey filaments (3.92, 5.88, 9.80, 19.60, 39.20, 58.80, 78.40 and 147.00 mN, Stoelting, Wood Dale, IL; equivalent in grams to 0.4, 0.6, 1.0, 2.0, 4.0, 6.0, 8.0 and 15.0) as our previous report [14]. The 50% withdrawal threshold was determined by using the up-down method [34]. In brief, rats were placed under transparent plastic boxes (28 X 10 X 10 cm) on a metal mesh floor (3 X 3 mm mesh). They were then left alone for at least 20 min of acclimation before sensory testing began. Testing was initiated with the filament which bending force was 19.60 mN, in the middle of the series. Von Frey filament applied to the plantar surface of each hind paw, and the most sensitive spot of the hind paw was first determined by probing various areas with the 19.60 mN filament. Stimuli were applied for 3-4 s to each hind paw while the filament was bent and were presented at intervals of several seconds. A brisk hind paw withdrawal to von Frey filament application was regarded as a positive response.

Heat sensitivity was assessed according to the Hargreaves method [35] to determine paw withdrawal latency (PWL) in response to a radiant heat (Model 390, IITC Life Science Inc. Woodland Hills, CA). *A* *radiant* heat source under the glass table was focused on center of the plantar surface. The heat intensity was set to produce PWL of approximately 10 s in normal animals, and the cut-off time was set at 20 s to prevent tissue damage as in previous reports [36,37]. Three times of heat stimuli were given for each paw at an interval of 5-10 min. The mean of PWL for three trials was taken for each paw of each rat.

### Basso-Beattie-Bresnahan (BBB) tests

For testing of hindlimb locomotor function, open-field locomotion was evaluated by using the 21-point BBB locomotion scale by trained investigators who were blind as to the experimental conditions as described [38]. BBB is a 22-point scale (scores 0–21) that systematically and logically follows recovery of hindlimb function from a score of 0, indicative of no observed hindlimb movements, to a score of 21, representative of a normal ambulating rodent.

### Drug administration

JNK inhibitor, SP600125 (Merk Calbiochem, Darmstadt, Germany) were dissolved in normal saline containing 2% DMSO and SP600125 (5 µg/rat) were administered intrathecally with 5 µl on POD 31. Since our preliminary study showed that a dose of 5 µg of SP600125 was optimal dose for analgesic effect in SCI-induced NP as reported [9], we used 5 µg/rat of SP600125 throughout experiments. For intrathecal injection, we used direct lumbar puncture as previously described [14,39,40]. In brief, experimental animals were anesthetized with 4% isoflurane in a mixture of O_2_ gas. The needle is inserted into the tissue to one side of the L5 or L6 spinous process so that it slips into the groove between the spinous and transverse processes. The tip of the needle is inserted so that approx. 0.5 cm is within the vertebral column. Identification of the needle in the intrathecal space was based on the presence of a sudden lateral tail movement that occurred after penetration of the ligamentum flavum. Once the needle was in the intrathecal space, a dose of drug was injected slowly for 10 s. As a vehicle control, normal saline containing 2% DMSO was injected during the same time points in separate injured animals.

### Tissue preparation

At POD 31, one hour after the treatment with AP, simulated AP, SP600125 or vehicle, rats were anesthetized with chloral hydrate (500 mg/kg) and perfused via cardiac puncture initially with 0.1 M phosphate buffered saline (PBS, pH 7.4) and subsequently with 4% paraformaldehyde in PBS. L4-L5 segments of spinal cord were dissected out, post-fixed by immersion in the same fixative for 2 h and placed in 30% sucrose in PBS. The segment was embedded in OCT for frozen sections as previously described [29], and cross sections were then cut at 10 µm on a cryostat (CM1850; Leica, Wetzlar, Germany). For molecular work, animals were perfused with 0.1 M PBS and segments of spinal cord (L4-L5) were isolated and frozen at -80°C.

### Immunohistochemistry

Tissue sections were incubated in 3% hydrogen peroxide in PBS for 10 min at room temperature (RT) to inhibit endogenous peroxidase activity. After washing with Tris-buffered saline including 0.1% Triton X-100(TBST), the sections were immersed in 5% normal serum (Vector Laboratories INC, Burlingame, CA) in TBST for 1 h at RT to block non-specific binding. They were then incubated with a rabbit anti-p-JNK (1:100; Cell Signaling Technology, Danvers, MA) or a rabbit anti-p-c-Jun (1:100; Cell Signaling Technology) overnight at 4°C, followed by biotinylated secondary antibodies (Dako, Carpinteria, CA). The ABC method was used to detect labeled cells using a Vectastain kit (Vector Laboratories INC). DAB served as the substrate for peroxidase. Some sections stained for p-JNK and p-c-Jun were double-labeled using specific antibody for identifying astrocytes (GFAP; 1:10,000; Millipore, Billerica, MA). For double labeling, FITC or Cy3-conjugated secondary antibodies (Jackson Immuno Research, West Grove, PA) were used. Nuclei were also labeled with DAPI according to the protocol of the manufacturer (Molecular Probes, Eugene, OR). In all controls, reaction to the substrate was absent if the primary antibody was omitted or replaced by a non-immune, control antibody. The immunofluorescent sections were mounted with Vectashield mounting medium (Vector). Fluorescence labeled signal was detected by a fluorescence microscope (Olympus), and capture of images and measurement of signal co-localization was performed with MetaMorph.

### Western blot

At POD 31, one hour after the treatment with AP, simulated AP and vehicle, whole lysates from L4-L5 segments of spinal cord were prepared as previously described [14]. Protein sample (40 µg) was separated on SDS-PAGE and transferred to nitrocellulose membrane (Millipore). The membranes were blocked in 5% nonfat skim milk or 5% bovine serum albumin in TBST for 1 h at room temperature followed by incubation with antibodies against p-JNK (1:3,000; Cell Signaling Technology), JNK (1:3,000; Cell Signaling Technology), p-c-Jun (1:1,000; Cell Signaling Technology), c-Jun (1:1,000; Santa Cruz Biotechnology, Santa Cruz, CA), GFAP (1:2,000; Millipore) and β-Tubulin (1:30,000; Sigma) at 4°C overnight. After washing, the membranes were incubated with HRP conjugated secondary antibodies (Jackson Immuno Research) for 1 h and immunoreactive bands were visualized by chemiluminescence using Supersignal (Thermo scientific Rockford IL). β-tubulin was used as an internal control. Relative intensity of each band to sham on Western blots was measured and analyzed by AlphaImager software (Alpha Innotech Corporation, San Leandro, CA). Background in films was subtracted from the optical density measurements. Experiments were repeated three times, and the values obtained for the relative intensity were subjected to statistical analysis.

### RNA isolation and RT-PCR

RNA was isolated using TRIZOL Reagent (Invitrogen, Carlsbad, CA) and 0.5 µg of total RNA was reverse-transcribed into first strand cDNA using MMLV according to the manufacturer’s instructions (Invitrogen). For PCR amplifications, the following reagents were added to 1 µl of first strand cDNA: 0.5 U taq polymerase (Takara, Kyoto, Japan), 20 mM Tris-HCl, pH 7.9, 100 mM KCl, 1.5 mM MgCl_2_, 250 µM dNTP, and 10 pmole of each specific primer. PCR conditions were as follows: denaturation at 94°C, 30 s, primer annealing at indicated temperature, 30 s, and amplification at 72°C, 30 s. PCR was terminated by incubation at 72°C for 7 min. The primers used for monocyte chemotactic protein-1 (MCP-1), macrophage inflammatory protein-1β (MIP-1β), MIP-3α and GAPDH were synthesized by the Genotech (Daejeon, Korea) and the sequences of the primers are as follows (5'–3'): MCP-1 forward, 5’-TCA GCC AGA TGC AGT TAA CG-3’; reverse, 5’-GAT CCT CTT GTA GCT CTC CAG C-3’ (94 bp, 61°C for 35 cycles); MIP-1β forward, 5’-TCC CAC TTC CTG CTG TTT CTC T-3’, reverse, 5’-GAA TAC CAC AGC TGG CTT GGA-3’ (106 bp, 60°C for 30 cycles); MIP-3α forward, 5’- GAC TGC TGC CTC ACG TAC AC’, CCL-20 reverse, 5’-CGA CTT CAG GTG AAA GAT GAT AG-3’; (120 bp, 60°C for 35 cycles); GAPDH forward, 5’- TCC CTC AAG ATT GTC AGC AA-3’; GAPDH, reverse, 5’- AGA TCC ACA ACG GAT ACA TT-3’ (308 bp, 50°C for 25 cycles). The plateau phase of the PCR reaction was not reached under these PCR conditions. After amplification, PCR products were subjected to a 1.5% agarose gel electrophoresis and visualized by ethidium bromide staining. The relative density of bands (relative to sham value) was analyzed by the AlphaImager software (Alpha Innotech Corporation). Experiments were repeated three times and the values obtained for the relative intensity were subjected to statistical analysis. The gels shown in figures are representative of results from three separate experiments.

### Statistical analysis

All data were collected by experimenters blinded to the surgery and reagent treatments and statistical analyses were done by using SPSS 15.0 (SPSS Science, Chicago, IL). In this study, we primarily decided the size of groups by power analysis using G*Power 3. Data except behavior tests are presented as the mean ± SD values and behavioral data are presented as the mean ± SEM. Comparison in between experimental groups was evaluated for statistical significance using unpaired Student’s t test. Multiple comparisons between groups were performed one-way ANOVA. Some behavioral scores were analyzed by repeated measures ANOVA. Dunnett’s case-comparison was used as Post hoc analysis. Statistical significance was accepted with *p* < 0.05.

## Results

### Acupuncture relieves neuropathic pain developed after SCI

We first examined whether chronic neuropathic pain (NP) is developed after SCI. The hindlimbs were paralyzed immediately after injury, and the rats recovered spontaneously extensive movement of hindlimbs within postoperative days (POD) 14 (Figure 1a). On responses to innoxious, mechanical stimuli, injured rats were not responsive in cut-off level to mechanical stimuli up to POD 7, and thereafter, mechanical PWT decreased progressively (Figure 1b). On responses to noxious, thermal stimuli, injured rats showed longer latency on POD 1 to POD 14 than sham control group and thereafter, decreased gradually (Figure 1c). Since the motor function is damaged until 14 d post-spinal cord injury, the higher values of PWL may be due to the loss of motor function. Then, significant NP from POD 14 for mechanical pain and POD 21 for thermal pain began to develop (Figure 1b, c) as reported [14]. By POD 28, animals recovered considerable motor function (BBB: 9.2 ± 0.1). With these scores, the rat is able to plantar placement of the paw with weight support in stance only or weight supported dorsal stepping and no plantar stepping. As our previous report [14], the injured rats displayed mechanical allodynia and thermal hyperalgesia at POD 28 (SCI group: PWT; 2.1 ± 0.2 g, PWL; 5.7 ± 0.1 s, vs. sham group: PWT; 15.0 ± 0.0 g; PWL; 10.3 ± 0.4 s). Both PWT and PWL in sham were not significantly different as compared to normal control (Normal, PWT: 15.0 ± 0.0 g; PWL: 10.1 ± 0.5 s).

**Figure 2:**
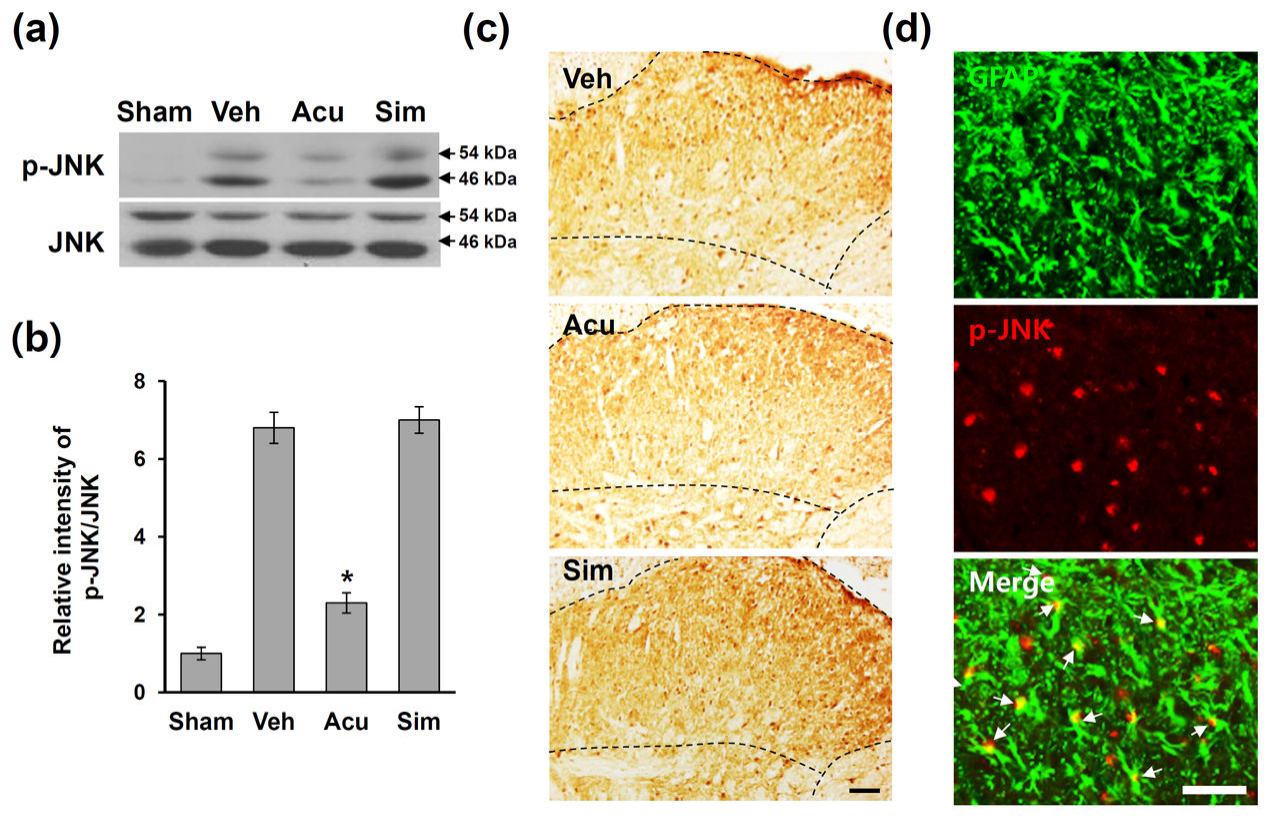
Acupuncture inhibits JNK activation after SCI. At POD 31, 1 h after AP treatment, lumbar (L4-L5) spinal tissues were isolated and total lysates or frozen tissue sections were prepared as described in the Methods section (n = 4). (a) Western blots of p-JNK. (b) Quantitative analyses of Western blots show that AP treatment significantly inhibited JNK activation when compared with that in vehicle or simulated AP control. Data are presented as mean ± SD (*p < 0.05 vs vehicle, df = 3, one-way ANOVA). (c) Immuohistochemistry of p-JNK. Dotted line indicates p-JNK-positive cells in lamina I and II of dorsal horn following SCI. (d) Double labeling showed that p-JNK immunoreactivity was co-localized in GFAP-positive astrocytes (arrows). Scale bars, 50 µm.

Next, we investigated the analgesic effects of AP on SCI-induced NP. As shown in Figure 1e, there were no significant differences in BBB scores in all groups after treatment. AP treatment significantly alleviated SCI-induced mechanical allodynia (PWT, 1 h AP: 11.3 ± 1.2 g vs. Pre: 2.1 ± 0.3 g, *p* < 0.01) (Figure 1f) and heat hyperalgesia (PWL, 1 h AP: 9.5 ± 0.7 s vs. Pre: 6.0 ± 0.5 s, *p* < 0.05) when compared with pre-treated value (Pre) (Figure 1g) as reported [14]. By contrast, simulated AP treatment showed no significant effects on PWT (2.5 ± 0.6 g) and PWL (5.7 ± 0.5s) as compared to pre-treated value (PWT, 2.5 ± 0.5 g; PWL, 6.0 ± 0.5 s) (Figure 2f, g). When we determined whether the restrain might induce stress-related analgesic effects, both PWT and PWL were not different between restrain and non-restrain animals as our previous report (data not shown) [14]. These results indicate that the restrain condition used in the present study did not influence on the analgesic effects by AP.

### Acupuncture inhibits JNK activation in astrocytes after SCI

JNK is known to be activated in astrocytes in the spinal cord after nerve injury [9,17] and to play an important role in NP sensitization [9,18]. However, the activation profile of JNK in the spinal cord, particularly in the dorsal horn during SCI-induced below level pain has not been determined. To examine the effects of AP on JNK activation in the L4-L5 spinal cords, Western blot analysis for p-JNK was performed. At 31 days after SCI, the level of p-JNK markedly increased as compared to sham control, and AP treatment decreased the level of p-JNK (Figure 2a). Quantitative analysis showed that AP significantly decreased the level of p-JNK when compared with vehicle or simulated AP treated groups (vehicle group: 6.8 ± 0.4; AP group: 2.3 ± 0.26; simulated AP group: 7.0 ± 0.34, *p* < 0.05) (Figure 2b). Immunocytochemistry revealed that after SCI, the number of p-JNK-immunoreactive cells was increased, and the p-JNK-positive cells were mainly observed in the superficial lamina including lamina I–II of the L4-L5 spinal dorsal horn (Figure 2c, Veh), while a very low p-JNK immunoreactivity was observed in sham control (data not shown). It is known that the laminae I–II layers of the spinal dorsal horn where the majority of unmyelinated Aδ and C fibers are involved in nociceptive signal processing and large-myelinated Aβ fibers terminated (shown dotted areas in Figure 2c). The p-JNK immunoreactivity in the superficial lamina was markedly reduced in AP-treated groups when compared with the vehicle or simulated AP-treated group (Figure 2c). Furthermore, double labeling showed that many p-JNK-positive cells were positive for GFAP, suggesting that p-JNK is expressed mainly in astrocytes (Figure 2d). Also, a small number of neurons were positive for p-JNK, but p-JNK-positive microglia were not observed (data not shown). Thus, these data suggested that AP may inhibit JNK activation primarily in astrocytes in the L4-L5 spinal dorsal horn after SCI.

### Acupuncture inhibits c-Jun activation after SCI

**Figure 3:**
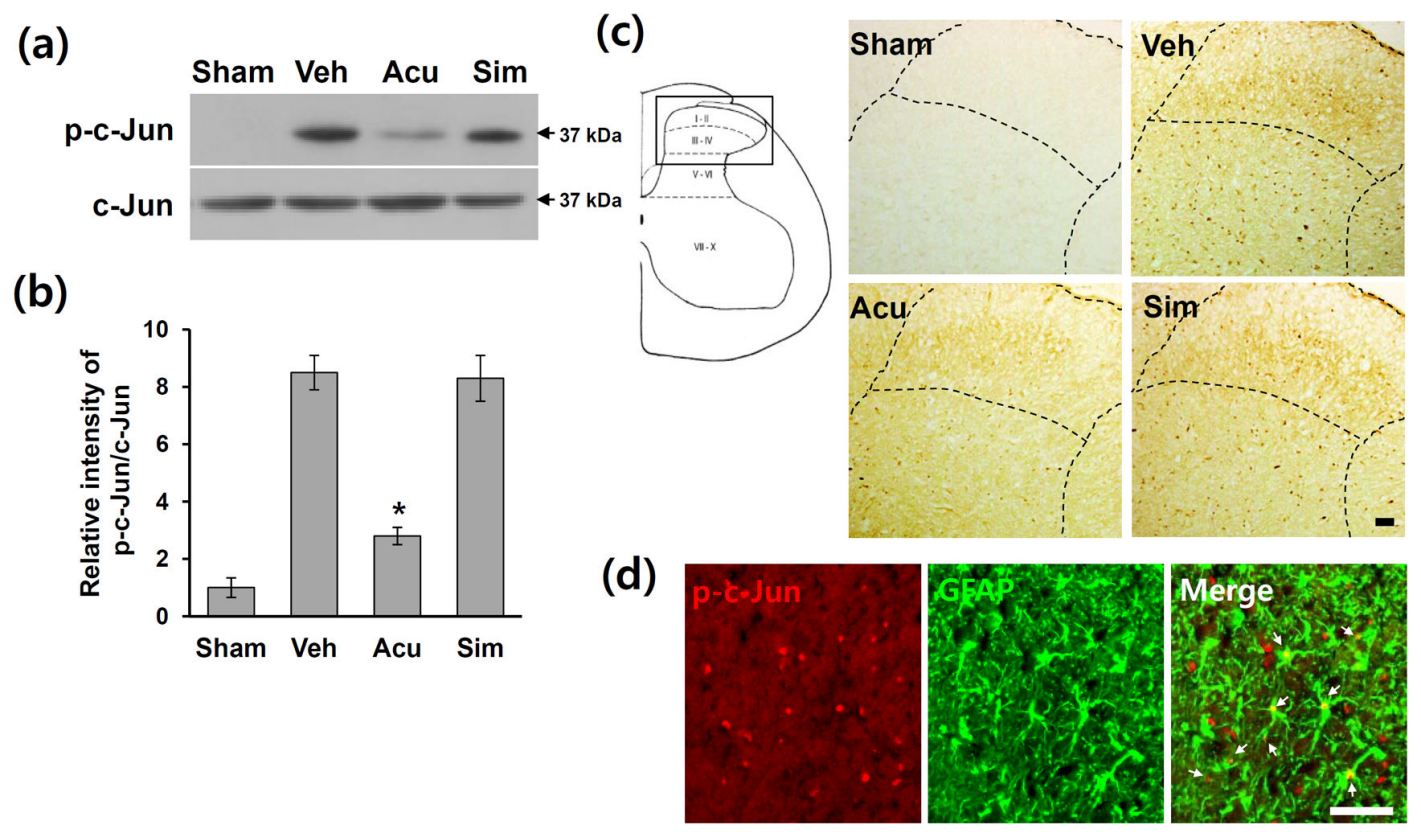
Acupuncture decreases the level of p-c-Jun after SCI. At 1 h after AP treatment, lumbar (L4-L5) spinal tissues were prepared and assessed by Western blot and immunohistochemistry (n = 4). (a) Western blots of p-c-Jun. (b) Quantitative analysis of Western blots showed that AP treatment significantly inhibited the level of p-c-Jun when compared with that in vehicle or simulated AP control. Data are presented as mean ± SD (*p < 0.05 vs vehicle, df = 3, one-way ANOVA). (c) Immunohistochemistry of p-c-Jun immunoreactvity in the lamina I and II. (d) Double labeling showed that p-c-Jun immunoreactivity was co-localized in GFAP-positive astrocytes (arrows). Scale bars, 50 µm.

The transcription factor, c-Jun, is a well-known as a substrate for JNK [41]. It has been shown that sciatic nerve ligation (SNL) induces c-Jun phosphorylation in astrocytes in the spinal cord, which is suppressed by a JNK inhibitor [9]. Therefore, we postulated that AP would inhibit c-Jun phosphorylation after SCI. Western blotting using an antibody against p-c-Jun was performed on total extracts from L4-L5 lumbar spinal cord treated with AP, simulated AP and vehicle. On post-operated day (POD) 31, the level of p-c-Jun was markedly increased as compared to sham control (Figure 3a). In addition, the level of p-c-Jun was significantly reduced in the AP-treated group when compared with vehicle control (vehicle group: 8.5 ± 0.6; AP group: 2.8 ± 0.6, *p* < 0.05) (Figure 3a, b). However, simulated AP treatment showed no effect on the level of p-c-Jun (simulated AP group: 8.3 ± 0.8) (Figure 3a, b). Immunohistochemistry also revealed that the intensity of p-c-Jun immunoreactivity increased markedly in the L4-L5 spinal dorsal horn after SCI (Figure 3c, Veh), while no immunoreactivity of p-c-Jun was observed in sham control (Figure 3c, Sham). Dotted lined areas indicate higher power views of the laminae I and II as shown in the left drawing figure. Also, there was a little change in the intensity of p-c-Jun immunoreactivity in other areas (minus dorsal horn) of L4-L5 spinal cord in vehicle-treated group as compared to acupuncture-treated group after injury (Figure 3c). AP treatment decreased the intensity of p-c-Jun immunoreactivity in the lamina I and II when compared with the vehicle or simulated AP-treated group (Figure 3c). Furthermore, double labeling showed that p-c-Jun-positive cells were mostly expressed in GFAP-positive astrocytes (arrows) in the dorsal horn area (Figure 3d), while few p-c-Jun-positive neurons and microglia were also observed (data not shown). Thus, these data indicate that AP inhibited c-Jun phosphorylation in astrocyte in the L4-L5 spinal dorsal horn after SCI.

### Acupuncture inhibits SCI-induced activation of astrocyte in the spinal cord dorsal horn

**Figure 4:**
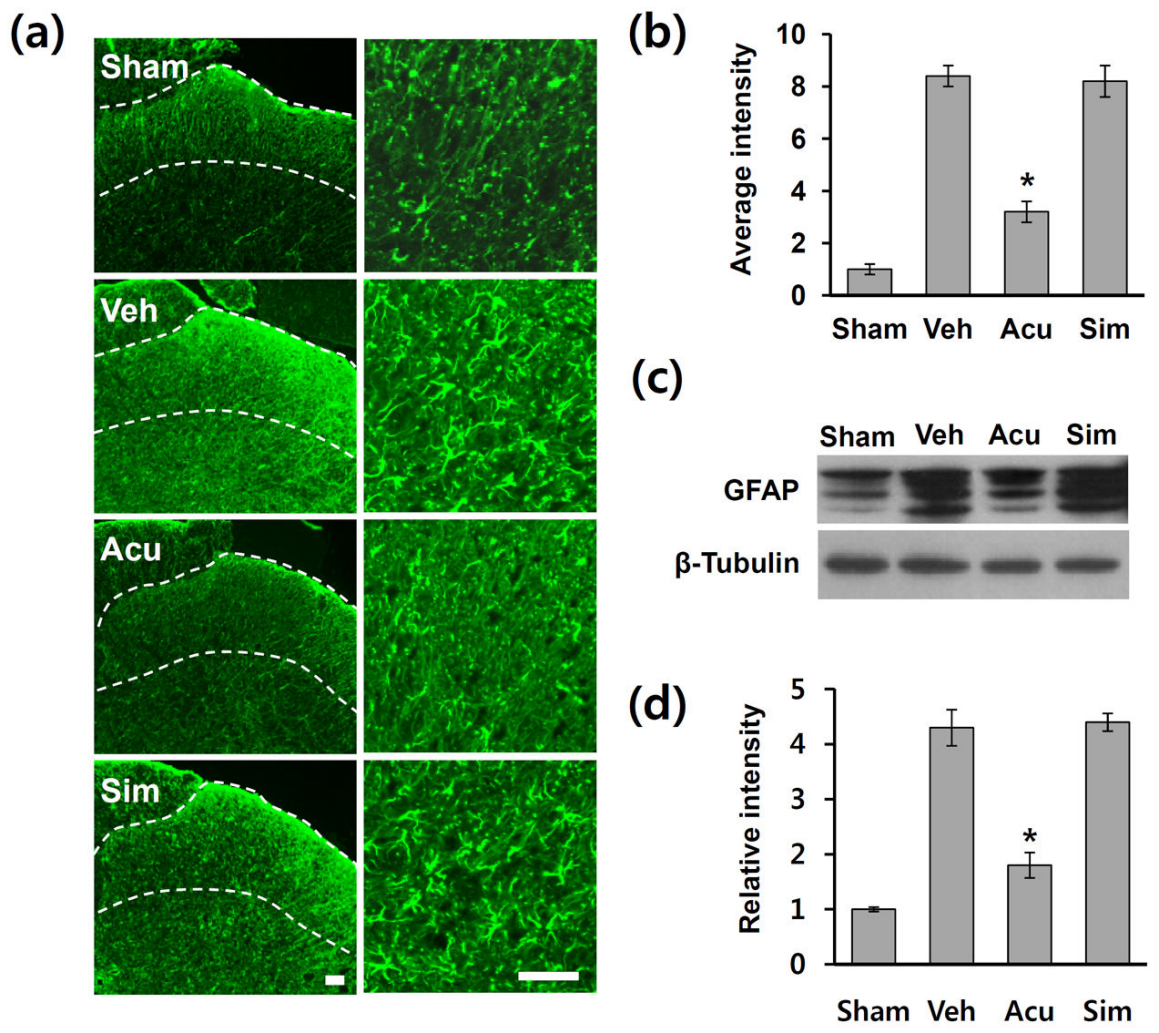
Acupuncture inhibits astrocyte activation after SCI. At 1 h after AP treatment, lumbar (L4-5) spinal tissues were isolated (n = 4). (a) Representative photographs of GFAP immunostaining in the dorsal horn (superficial layer) indicated by dotted lines on POD 31. Scale bars, 50 µm. (b) Densitometric analysis reveals that GFAP-immunoreactivity was dramatically increased in the dorsal horn of injured spinal cord and AP treatment significantly reduced the GFAP immunoreactivity as compared to vehicle control. (c) Western blots of GFAP. (d) Quantitative analysis of Western blots showed that AP treatment significantly inhibited GFAP expression when compared with vehicle control. All data are presented as mean ± SD (*p < 0.05 vs vehicle, df = 3, one-way ANOVA).

Activation of astrocytes in the spinal dorsal horn after nerve injury and spinal hemisection has been implicated in NP [9,17,42,43]. Since the increased intensity of GFAP in astrocytes is well known to be used as a marker for their activation [44], and AP inhibits JNK activation in astrocytes in L4-L5 dorsal horn after SCI (Figure 2), we hypothesized that AP would inhibit astrocyte activation after injury. Therefore, we performed GFAP immunostaining on L4-L5 spinal cord sections of animals treated with sham, vehicle, AP, and simulated AP. After SCI, the intensity of GFAP immunoreactivity was markedly increased and mostly observed in superficial lamina including lamina I–II of the L4-L5 spinal dorsal horn as reported [42] (Figure 4a, Veh), while very low GFAP immunoreactivity was observed in the sham control (Figure 4a, Sham). One hour after AP treatments, the intensity of GFAP immunoreactivity in the dorsal horn was markedly decreased in AP-treated groups when compared with the vehicle or simulated AP-treated group (Figure 4a). Densitometric analysis revealed that fluorescent intensity in AP-treated group was significantly lower than that in vehicle or simulated AP control (Figure 4b). Western blotting using an antibody against GFAP was performed on total extracts from L4-L5 lumbar spinal cords from sham, vehicle-, AP- and simulated AP-treated groups on POD 31. Parallel with the immunohistochemistry, the level of GFAP increased after injury as compared to sham control and significantly reduced in the AP-treated group when compared with vehicle control (vehicle group: 4.3 ± 0.33; AP group: 1.8 ± 0.23, *p* < 0.05) (Figure 4c, d). However, simulated AP treatment did not affect the level of GFAP (simulated AP group: 4.4 ± 0.16) (Figure 4c, d). Thus, these data indicate that AP inhibited astrocyte activation in the L4-L5 spinal dorsal horn after SCI.

### Analgesic effect of acupuncture is mediated through inhibition of JNK activation in astrocytes after SCI

**Figure 5:**
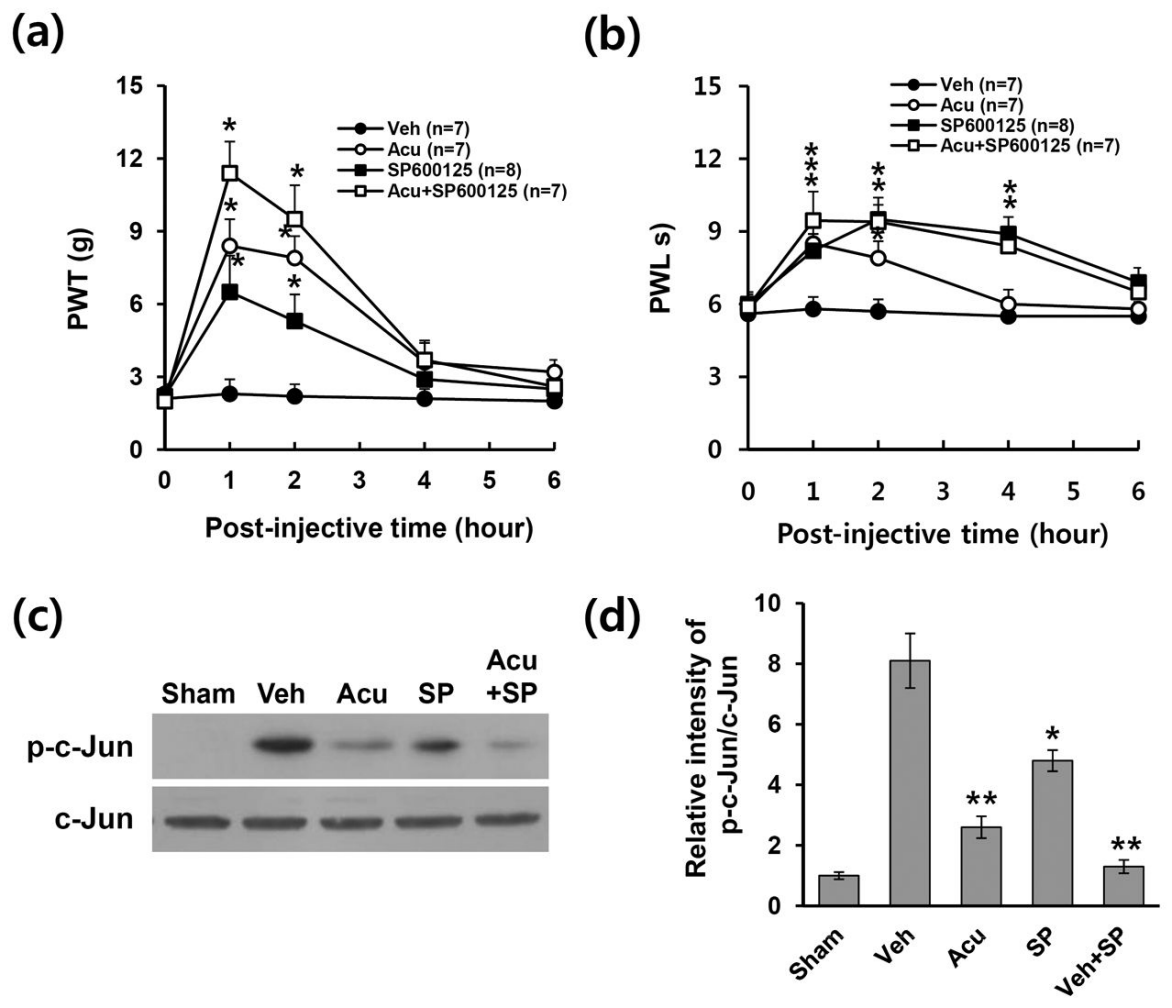
Intrathecal administration of JNK inhibitor inhibits neuropathic pain. SP600125 (5 µg/rat), a specific JNK inhibitor, was injected intrathecally (5 µl) at POD 31 as described in the Methods section (n = 7). SP600125 treatment significantly relieved SCI-induced mechanical allodynia (a) and heat hyperalgesia (b) when compared with those in vehicle control. Data (a-b) are presented as mean ± SEM (*p < 0.05, df = 3, repeated measures ANOVA). (c) Western blots of p-c-Jun at 1 h after treatment of AP or SP600125 (n = 4). (d) Quantitative analysis of Western blots showed that SP600125 treatment significantly decreased the level of p-c-Jun when compared with vehicle control. All data are presented as mean ± SD (*p < 0.05, **p < 0.01 vs vehicle, df = 4, one-way ANOVA).

To determine whether JNK activation would play a role in the pain sensitization after SCI, SP600125, a specific JNK inhibitor, was delivered intrathecally into L4/5 spinal cord via direct lumbar puncture once on POD 31. Administration of SP600125 (5 μg) significantly increased the mechanical PWT and thermal PWL as compared to vehicle control and peak at 1 h after post-injection (SP600125 group: PWT; 6.5 ± 1.5 g and PWL; 8.2 ± 0.7 s vs. vehicle group: PWT; 2.3 ± 0.6 g and PWL; 5.8 ± 0.5 s, p < 0.05) (Figure 5a, b). This result suggested that JNK activation in the dorsal horn at L4-L5 may mediate SCI-induced NP at below level. Furthermore, co-treatment with AP and SP600125 led to more significant increases in mechanical PWT (SP600125 + AP: 11.4 ± 1.3 g; SP600125 alone: 6.5 ± 1.5 g; AP alone: 8.4 ± 1.1 g, p < 0.05) and PWL (SP600125 + AP: 9.45 ± 1.2 s; SP600125 alone: 8.2 ± 0.7 s; AP alone: 8.5 ± 0.7 s, p < 0.05) when compared with AP alone or SP600125 alone group (Figure 5a, b). Thus, AP and SP600125 co-treatment appeared to be additive effects on pain relief. At 1 h after treatment, SP600125 treatment significantly reduced the level of p-c-Jun when compared with vehicle control and co-treatment of SP600125 and AP appeared to be additive effects on c-Jun phosphorylation (vehicle: 8.1 ± 0.9; AP alone: 2.6 ± 0.4; SP600125 alone: 4.8 ± 0.4; SP600125 + AP: 1.3 ± 0.2, p < 0.05) (Figure 5c, d). These results suggest that the analgesic effect of AP is likely mediated in part by inhibiting JNK and c-Jun activation in astrocytes after SCI.

### Acupuncture inhibits JNK-dependent MCP-1, MIP-1β, and MIP-3α expression after SCI

**Figure 6:**
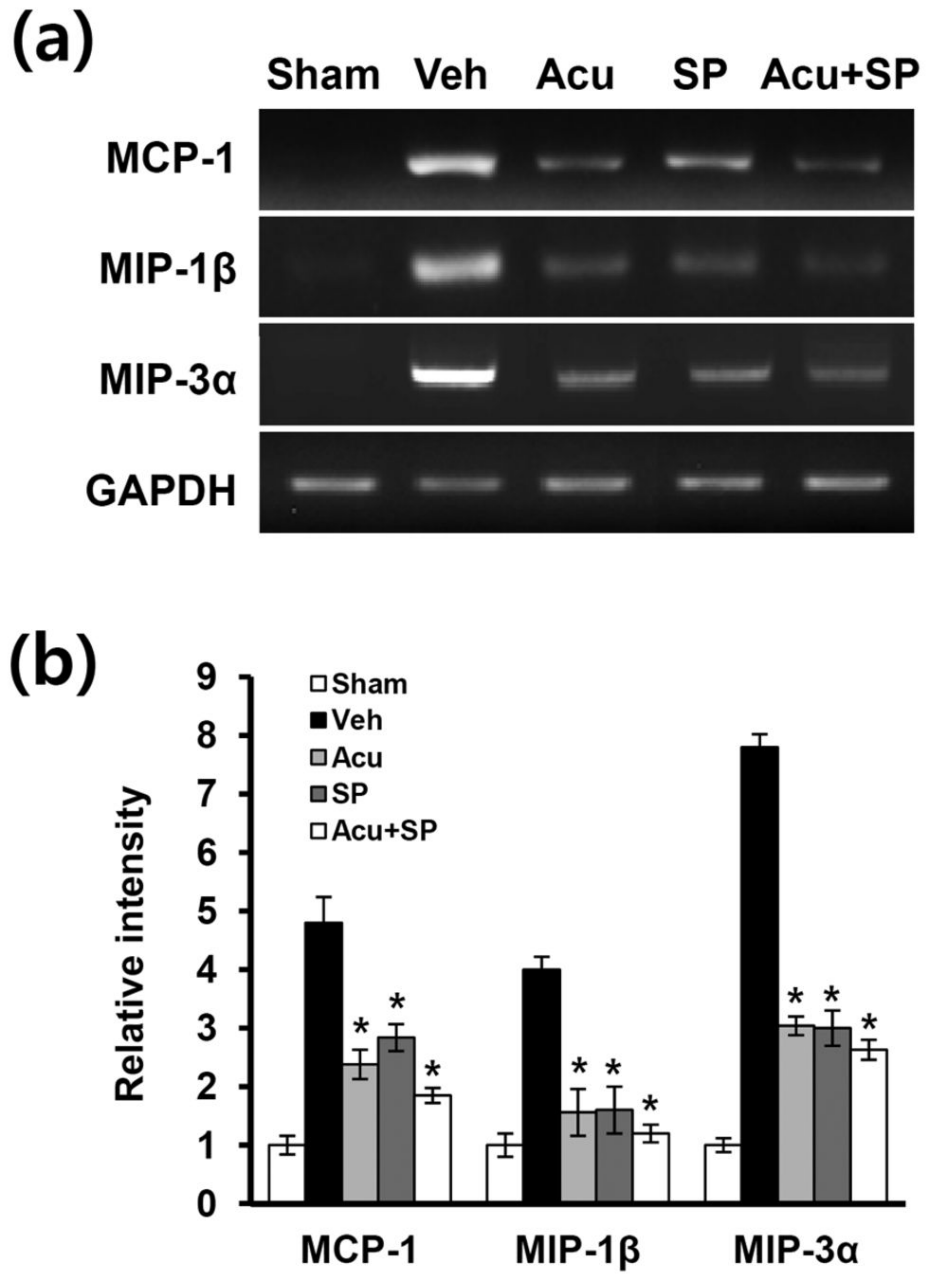
Acupuncture inhibits the expression of JNK-dependent chemokines after SCI. At 1 h after treatment with AP or SP600125 (5 µg/rat) at POD 31, total RNA from spinal cords were prepared as described in the Methods section (n = 4). (a) RT-PCR of MCP-1, MIP-1β, and MIP-3α mRNA after injury. (b) Quantitative analysis of RT-PCR showed that AP or SP600125 treatment significantly inhibited the expression of chemokines when compared with vehicle-treated control. All data are presented as mean ± SD (*p < 0.05 vs vehicle, df = 4, one-way ANOVA).

JNK pathway is known to be involved in chemokines expression such as MIP-1, MIP-1β, and MIP-3α [45–47]. These chemokines are also known to be produced by activated astrocytes [48]. Furthermore, recent evidence shows that MCP-1 chemokine is up-regulated in spinal astrocytes via JNK pathway after sciatic nerve ligation (SNL) and contributes to NP development [16]. Therefore, we postulated that chemokines MCP-1, MIP-1β, and MIP-3α would be expressed JNK-dependently in lumbar spinal cord after SCI and AP would inhibit these chemokines expression. RT-PCR analysis revealed that at the expression of MCP-1, MIP-1β, and MIP-3α mRNA markedly increased at 31 d after SCI and significantly decreased by AP or SP600125 (Figure 6a, b) at 1 h after treatment. Furthermore, simultaneous treatment of AP and SP600125 more decreased the levels of MIP-1, MIP-1β, and MIP-3α mRNA expression when compared with AP alone or SP600125 alone group (Figure 6a, b). These results suggest that the analgesic effect of AP may be mediated in part by inhibiting JNK-dependent MIP-1, MIP-1β, and MIP-3α expression after injury.

## Discussion

Our recent study shows that AP inhibits SCI-induced below level pain by inhibiting ROS production and microglial activation via inhibition of p38MAPK and ERK activation in microglia [14]. The present study demonstrated an additional mechanism of analgesic action of AP after injury. Our results also showed that JNK was markedly activated in astrocytes at L4-L5 spinal cord dorsal horn at 31 d after SCI. AP treatment inhibited the activation of JNK and phosphorylation of c-Jun, a well-known substrate for JNK. Furthermore, we demonstrated that JNK activation in spinal astrocytes at delayed time after SCI appeared to be essential for the sensitization of NP by demonstrating the inhibitory effect of a specific JNK inhibitor (SP600125) on mechanical allodynia and heat hyperalgesia. Furthermore, the expression of chemokines such as MIP-1, MIP-1β, and MIP-3α, which is known to be involved in injury-induced NP, was significantly attenuated by AP and the JNK inhibitor. Taken together, our results thus indicate that the analgesic effect of AP is likely mediated in part by inhibiting the activation of JNK/c-Jun pathway in activated astrocytes after injury.

In the present study, we showed both mechanical allodynia and thermal hyperalgesia were significantly alleviated by AP applied at GV26 and GB34 simultaneously. In our previous report, the two acupoints, GV26 and GB34, were identified as the most neuroprotective acupoints after injury (total 7 different acupoints tested). Furthermore, we found that AP applied simultaneously at GV26 and GB34 acupoints was more effective than a separate stimulation at each acupoint [30]. In addition, NP after SCI was also significantly alleviated by simultaneous AP at GV26 and GB34 by inhibiting microglia activation [14,28]. Thus, we choose simultaneous AP at GV26 and GB34 in this study, although there was an analgesic effect of each acupoint.

As a member of the MAPK family, JNK has been known to play a critical role in intracellular signal transduction. Both JNK1 and JNK2 are ubiquitously expressed, while JNK3 is expressed primarily in the nervous system, endocrine pancreas, and heart [49,50]. After SCI, JNK activation has been known to induce secondary injury and limits motor recovery [21,22]. In particular, JNK3 is known to be involved in oligodendrocytes cell death after SCI [19,20]. However, the role of JNK activation in SCI-induced NP developed at delayed time after injury has not been examined. Our results showed that the phosphorylation of both JNK1 and JNK2 was highly up-regulated and mainly observed in astrocytes in L4-L5 on POD 31 after SCI (See Figure 2). However, Zhuang et al. [9] reports that only phosphorylated JNK1 is increased in astrocytes after sciatic nerve ligation (SNL) although both JNK1 and JNK2 are constitutively expressed in the spinal cord. The discrepancy in different phosphorylation of JNK isoforms may be attributable to the type of injuries (peripheral versus central nerve injury).

Several studies indicate that microglia also plays a critical role in SCI-induced NP development [6,8]. However, the role of astrocytes on NP following CNS injuries such as SCI has not been fully examined although recent evidence suggests astrocytes may modulate neuronal hyperexcitability in spinal hemissection model [42]. Several reports also show that pheripheral nerve injury such as SNL induces activation of JNK pathway in astrocytes in the spinal cord [7,9,17,51]. In addition, administration of a JNK inhibitor suppresses activation of astrocytes and reduces SNL-induced NP [9,18]. Furthermore, the role of astrocytes in maintaining NP is further supported by demonstrating the reversal of mechanical allodynia after intrathecal infusion of L-α-AA, a cytotoxin specific for astrocytes [9]. Treatment with propentofylline, a methylxanthine derivative, attenuates mechanical allodynia and thermal hyperalgesia by inhibiting astrocytes activation in spinal cord dorsal horn after spinal hemisection injury [42]. Our study demonstrated that the levels of p-JNK and p-c-Jun were increased at POD31 and most p-JNK-positive and p-c-Jun-positive astrocytes were observed in the lumbar dorsal horn (see Figures 2, 3). In addition, astrocytes activation was significantly inhibited by AP treatment (see Figure 4). Furthermore, SCI-induced mechanical allodynia and thermal hyperalgesia were inhibited by SP600125, a specific JNK inhibitor, treatment (see Figure 5). Our results thus showed that JNK/c-Jun pathway in astrocytes plays an important role in pain development after SCI. To our knowledge this is the first study demonstrating the role of spinal astrocytes in CNS injury-induced chronic NP.

It is known that spinal glial cells enhance and maintain NP by releasing potent neuromodulators, such as pro-inflammatory cytokines and chemokines [52]. While the role of pro-inflammatory cytokines such as TNF-α, IL-1β, and IL-6 in NP sensitization has been reported [53–57], a very little information is currently available regarding the role of chemokines in NP development and/or maintenance. Various chemokines are known to be produced by activated astrocytes [48]. In addition, recent evidence indicates that JNK pathway is involved in the production of chemokines such as MIP-1, MIP-1β, and MIP-3α. For example, treatment with a JNK inhibitor inhibits production of CCL-2 (MCP-1) and CCL-4 (MIP-1β) in IL-1β- or TNF-α-stimulated trimester decidual cells [47]. JNK pathway is also involved in CCL-20 production in keratinocytes and Rheumatoid arthritis synoviocytes after inflammatory stimuli [45,46]. Furthermore, MCP-1 is produced by astrocytes via JNK-mediated pathway after SNL and involved in NP and central sensitization (hyperactivity of dorsal horn neurons) [16]. Furthermore, our results showed that the expression of MIP-1, MIP-1β, and MIP-3α were increased in L4-L5 spinal cord after SCI and inhibited by AP treatment (see Figure 6). We also showed that treatment with SP600125, a JNK inhibitor, inhibited the expression of MIP-1, MIP-1β, and MIP-3α (see Figure 6). Since JNK activation was observed mainly in astrocytes after SCI (See Figure 2), these results suggest that the analgesic effect of AP after SCI may be mediated in part by inhibiting MIP-1, MIP-1β, and MIP-3α production via JNK signaling in activated astrocytes. However, the role of chemokines such as MIP-1, MIP-1β, and MIP-3α in SCI-induced NP were not examined in the present study.

## Conclusions

We demonstrated that JNK activation in astrocytes plays a critical role on chronic NP at below level after SCI. Our results also showed that AP treatment significantly relieved the below-level pain following SCI by inhibiting astrocytes activation and JNK/p-c-Jun pathway in astrocytes at L4-L5 level after injury. Taken together with our recent report [14], our study demonstrated that analgesic effects of AP are likely mediated in part by inhibiting inflammatory responses via inhibition of MAPKs (p38, ERK, and JNK MAPK) in both activated microglia and astrocytes after SCI. Furthermore, the present study suggests an application of AP as an adjunct treatment for chronic NP in SCI patients.

## Supporting Information

Figure S1 Photograph showing an immobilization apparatus for acupuncture treatment without anesthesia.
(PDF)
