# Supplementary figures and images for: Analgesic Effect of Acupuncture Is Mediated via Inhibition of JNK Activation in Astrocytes after Spinal Cord Injury
Source: PLoS One. 2013 Sep 9;8(9):e73948. doi: 10.1371/journal.pone.0073948 (PMC3767587; doi:10.1371/journal.pone.0073948)

**Figure S1**

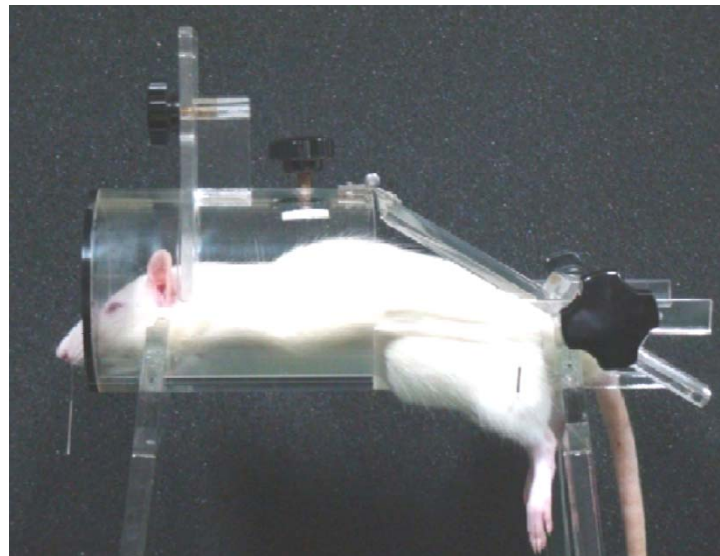

Supplement: Figure S1 — Photograph showing an immobilization apparatus for acupuncture treatment without anesthesia. (PDF) [file pone.0073948.s001.pdf]
